# Supplementary material for: Brain FGF2 and NCAM1 contribute to FGFR1-dependent progression of estrogen receptor-positive breast cancer brain metastases
Source: Nat Commun. 2026 May 28;17:6945. doi: 10.1038/s41467-026-73726-5 (PMC13388965; doi:10.1038/s41467-026-73726-5)
Supplement: Supplementary file 2 — Descriptions of Additional Supplementary Files [file 41467_2026_73726_MOESM2_ESM.pdf]

Descriptions of Additional Supplementary Files

Supplementary Data 1: Differential gene signature clusters from digital spatial profiling.

Supplementary Data 2: Downregulated genes from digital spatial profiling.

Supplementary Data 3: Differential gene signatures from RNA sequencing.

Supplementary Data 4: Differential gene signature clusters unique to FGF2 treatment.

Supplementary Data 5: Differential gene signature clusters shared with FGF2 and NCAM1 treatment.
