# Supplementary material for: Brain FGF2 and NCAM1 contribute to FGFR1-dependent progression of estrogen receptor-positive breast cancer brain metastases
Source: Nat Commun. 2026 May 28;17:6945. doi: 10.1038/s41467-026-73726-5 (PMC13388965; doi:10.1038/s41467-026-73726-5)
Supplement: Supplementary file 8 — Reporting Summary [file 41467_2026_73726_MOESM8_ESM.pdf]

nature portfolio

Corresponding author(s): Diana Cittelly

Last updated by author(s): May 15, 2026

Reporting Summary

Nature Portfolio wishes to improve the reproducibility of the work that we publish. This form provides structure for consistency and transparency in reporting. For further information on Nature Portfolio policies, see our Editorial Policies and the Editorial Policy Checklist.

Statistics

For all statistical analyses, confirm that the following items are present in the figure legend, table legend, main text, or Methods section.

n/a	Confirmed	
☐	☒	The exact sample size (n) for each experimental group/condition, given as a discrete number and unit of measurement
☐	☒	A statement on whether measurements were taken from distinct samples or whether the same sample was measured repeatedly
☐	☒	The statistical test(s) used AND whether they are one- or two-sided<br>*Only common tests should be described solely by name; describe more complex techniques in the Methods section.*
☒	☐	A description of all covariates tested
☐	☒	A description of any assumptions or corrections, such as tests of normality and adjustment for multiple comparisons
☐	☒	A full description of the statistical parameters including central tendency (e.g. means) or other basic estimates (e.g. regression coefficient) AND variation (e.g. standard deviation) or associated estimates of uncertainty (e.g. confidence intervals)
☐	☒	For null hypothesis testing, the test statistic (e.g. F, t, r) with confidence intervals, effect sizes, degrees of freedom and P value noted<br>*Give P values as exact values whenever suitable.*
☒	☐	For Bayesian analysis, information on the choice of priors and Markov chain Monte Carlo settings
☒	☐	For hierarchical and complex designs, identification of the appropriate level for tests and full reporting of outcomes
☒	☐	Estimates of effect sizes (e.g. Cohen's d, Pearson's r), indicating how they were calculated

Our web collection on statistics for biologists contains articles on many of the points above.

Software and code

Policy information about availability of computer code

Data collection	MRI was performed on a Bruker animal 9.4 Tesla BioSpec MRI scanner (Bruker Medical) equipped with a 1H receive-only 4-channel mouse brain surface array coil using Bruker ParaVision 360neo v3.3 acquisition software. In vivo bioluminescence (IVIS) was aquired on an IVIS Spectrum with Living Image version 4.8.2. Immunoblots were acquired on LiCor Odessey CLx with ImageStu with dio Version 5.0 and 6.0 software. qRT-PCR was performed on Applied Biosystems QuantStudio 6 Flex with QuantStudio Software v1.7.2. Organotypic co-culture images were acquired on an Olympus MVX10 fluorescent scope with cellSens Dimension v2.1 software. IHC images were obtained with Aperio ScanScope T3 (Leica Biosystems). Immunofluorescence images were acquired on a 3I Marianas spinning disk confocal microscope with SlideBook 6 software. Growth assays were performed using Incucyte Live Cell Imaging S3 with Incucyte v2023A software. Digital Spatial Transcriptomics and RNA sequencing was performed on an Illumina NovaSeq X-plus sequencer.
Data analysis	MR image analysis was performed using Bruker ParaVision 360neo v3.3 software. IVIS was analyzed using Living Image 2.60.1. Immunoblots were analyzed with ImageStudio Version 5.0 and 6.0 software. qRT-PCR was analyzed with QuantStudio Software v1.7.2. Organotypic co-culutre, migration, and immunofluorescence were analyzed with ImageJ v1.54. IHC analysis was performed with Aperio ImageScope Analysis Software (v12.4). Incucyte analysis was performed with Incucyte v2023A software. Digital spatial transcriptomics and RNA-sequencing data were analyzed with R v4.4.2 and v4.5. All other statistical analyses were performed with GraphPad Prism 10.4.2.

For manuscripts utilizing custom algorithms or software that are central to the research but not yet described in published literature, software must be made available to editors and reviewers. We strongly encourage code deposition in a community repository (e.g. GitHub). See the Nature Portfolio guidelines for submitting code & software for further information.

Data

Policy information about availability of data

All manuscripts must include a data availability statement. This statement should provide the following information, where applicable:

- Accession codes, unique identifiers, or web links for publicly available datasets
- A description of any restrictions on data availability
- For clinical datasets or third party data, please ensure that the statement adheres to our policy

RNA-sequencing and digital spatial transcriptomics data that support the findings of this study have been deposited in the National Center for Biotechnology Information Gene Expression Omnibus (GEO) and are accessible through the GEO Series accession numbers: GSE301400 and GSE301558. Source data is provided with this paper. All raw imaging data is available immediately upon request to the corresponding author.

Research involving human participants, their data, or biological material

Policy information about studies with human participants or human data. See also policy information about sex, gender (identity/presentation), and sexual orientation and race, ethnicity and racism.

Reporting on sex and gender	Studies only used de-identified human clinical brain metastases specimens for which we only had information regarding subtype of breast cancer. No information of sex and gender was accessed by the research team.
Reporting on race, ethnicity, or other socially relevant groupings	Studies only used de-identified human clinical brain metastases specimens for which we only had information regarding subtype of breast cancer. No information of race or ethnicity was accessed by the research team.
Population characteristics	Menopausal status and breast cancer subtype are noted.
Recruitment	Archival de-identified human brain metastases samples were obtained under secondary use Colorado Multiple Institutional Review Board (COMIRB) protocol approval.
Ethics oversight	Colorado Multiple Institutional Review Board (COMIRB) approved the use of these samples.

Note that full information on the approval of the study protocol must also be provided in the manuscript.

Field-specific reporting

Please select the one below that is the best fit for your research. If you are not sure, read the appropriate sections before making your selection.

☒ Life sciences ☐ Behavioural & social sciences ☐ Ecological, evolutionary & environmental sciences

For a reference copy of the document with all sections, see nature.com/documents/nr-reporting-summary-flat.pdf

Life sciences study design

All studies must disclose on these points even when the disclosure is negative.

Sample size	No statistical analysis was performed to determine sample size. Sample sizes were determined based on previous reports.
Data exclusions	No data were excluded.
Replication	All data shown in this manuscript was successfully replicated. Some animal experiments were performed once and reproducibility tested in secondary experiments that included the same experimental groups.
Randomization	For animal experiments involving injections of different cells, groups were littermate and age-matched and injections were blinded. For treatment of established tumors, groups were randomized then tested for statistical differences in tumor burden. If groups had significant differences in tumor burden, randomization was repeated.
Blinding	For in vivo experiments involving injections of different cells, injections were performed by an investigator who was blinded to the experimental conditions. Where possible, analyses were blinded to experimental conditions or performed using automated functions to reduce bias. Analyses of organotypic spheroid growth, immunofluorescence images, and histological quantification of metastases were blinded to experimental conditions or performed using automated functions.

Reporting for specific materials, systems and methods

We require information from authors about some types of materials, experimental systems and methods used in many studies. Here, indicate whether each material, system or method listed is relevant to your study. If you are not sure if a list item applies to your research, read the appropriate section before selecting a response.

Materials & experimental systems

n/a	Involved in the study
☐	☒ Antibodies
☐	☒ Eukaryotic cell lines
☒	☐ Palaeontology and archaeology
☐	☒ Animals and other organisms
☒	☐ Clinical data
☒	☐ Dual use research of concern
☒	☐ Plants

Methods

n/a	Involved in the study
☒	☐ ChIP-seq
☒	☐ Flow cytometry
☐	☒ MRI-based neuroimaging

Antibodies

Antibodies used

Primary Antibodies:
Host: Rabbit, Anti-FGFR1 antibody, abcam, cat. no: ab7646, clone: EPR806Y, lot: gr3180241-3, 1056244-2, WB 1:1000;
Host: Rabbit, Anti-FGFR1 (phospho Y654) antibody, abcam, cat. no: ab59194, clone: polyclonal, lot: gr3326759-7, 1082749-1, IF 1:400, IHC 1:800;
Host: Rabbit, Phospho-Akt (Ser473), Cell Signaling, cat. no: 4060S, clone: D9E, lot: 25, WB 1:1000;
Host: Rabbit, Akt Antibody , Cell Signaling, cat. no: 9272S, clone: polyclonal, lot: 28, WB 1:1000;
Host: Rabbit, p44/42 MAPK (Erk1/2) Antibody, Cell Signaling, cat. no: 9102S, clone: polyclonal, lot: 28, WB 1:1000;
Host: Rabbit, Phospho-p44/42 MAPK (Erk1/2) (Thr202/Tyr204) , Cell Signaling, cat. no: 9101S, clone: polyclonal, lot: 27, WB 1:1000, IF 1:200;
Host: Goat, Human/Mouse NCAM-1/CD56 Antibody, R&D, cat. no: AF2408, clone: polyclonal, lot: VOK0222081, WB 1:500, IF: 1:200;
Host: Mouse, GAPDH Mouse mAb, Cell Signaling, cat. no: 97166S, clone: D4C6R, lot: 7, WB 1:2000;
Host: Mouse, Anti-TUBA4A (TUBA1) Antibody, Sigma, cat. no: T5168, clone: B-5-1-2, lot: 035M4878V, WB 1:1000;
Host: Rabbit, FGFR1 Polyclonal Antibody, Invitrogen, cat. no: PA5-25979, clone: polyclonal, lot: wc3214267, IHC: 1:400;
Host: Rat, GFAP Monoclonal Antibody, Invitrogen, cat. no: 13-0300, clone: 2.2B10, lot: YI37984, IF 1:200;
Host: Rabbit, FGF basic/FGF2/bFGF Antibody, Novusnio, cat. no: NBP3-15367, clone: 8W6M5, lot: 4000000618, IF 1:100;
Host: Mouse, Anto-Human Cytokeratin, DAKO, cat. no: M0821, clone: MNF116, lot: 20056932, IF 1:100;
Host: Guinea pig, Synapsin1/2 antibody, Synaptic Systems, cat. no: 106004, clone: polyclonal, lot: 2-28, IF 1:1000;
Host: Mouse, PSD-95 Monoclonal Antibody, Invitrogen, cat. no: MA1-045, clone: 6G6-1C9, lot: ZD392083, IF 1:500;
Host: Rabbit, Anti Iba1, Wako, cat. no: 019-19741, clone: polyclonal, lot: PTG5394, IF 1:100;
Host: Mouse, Anti-Mouse FGF2/bFGF Antibody, Antibody System, cat. no: P15655, clone: GAL-F2, lot: 251102;
Host: Rabbit, Anti-Neural Cell Adhesion Molecule Antibody, Sigma, cat. no: AB5032, clone: polyclonal, lot:419518;
Host: Mouse, IgG1 Isotype Control from murine myeloma, Sigma, cat. no: M5284, clone: MOPC21, lot: 109M4890V.
Secondary Antibodies:
Donkey anti-Mouse IgG (H+L) Highly Cross-Adsorbed Secondary Antibody, Alexa Fluor™ 790, Invitrogen, cat. no: A11371, lot: 2300923, WB 1:5000;
Goat anti-Rabbit IgG (H+L) Highly Cross-Adsorbed Secondary Antibody, Alexa Fluor™ 680, Invitrogen, cat. no: A21109, lot: 2260898, WB 1:5000;
Goat anti-Mouse IgG (H+L) Highly Cross-Adsorbed Secondary Antibody, Alexa Fluor™ 680, Invitrogen, cat. no: A21058, lot: 2115694, WB 1:5000;
Donkey anti-Goat IgG (H+L) Cross-Adsorbed Secondary Antibody, Alexa Fluor™ 680, Invitrogen, cat. no: A21084, lot: 2300936, WB 1:5000;
Donkey anti-Rabbit IgG (H+L) Highly Cross-Adsorbed Secondary Antibody, Alexa Fluor™ 790, Invitrogen, cat. no: A11374, lot: 2160395, WB 1:5000;
Alexa Fluor® 488 AffiniPure® Donkey Anti-Rat IgG (H+L), Jackson Immunoresearch, cat. no: 712-545-153, lot: 147289, IF 1:200;
Alexa Fluor® 594 AffiniPure® Donkey Anti-Rat IgG (H+L), Jackson Immunoresearch, cat. no: 712-585-153, lot: 148486 IF 1:200;
Cy™3 AffiniPure® Donkey Anti-Guinea Pig IgG (H+L), Jackson Immunoresearch, cat. no: 706-165-148, lot: 154462 IF 1:200;
Alexa Fluor® 488 AffiniPure® Donkey Anti-Mouse IgG (H+L), Jackson Immunoresearch, cat. no: 715-545-150, lot: 148532 IF 1:200;
Alexa Fluor® 594 AffiniPure® Donkey Anti-Mouse IgG (H+L), Jackson Immunoresearch, cat. no: 715-585-150, lot: 147525 IF 1:200;
Alexa Fluor® 647 AffiniPure® Donkey Anti-Mouse IgG (H+L), Jackson Immunoresearch, cat. no: 715-605-150, lot: 149689 IF 1:200;
Alexa Fluor® 594 AffiniPure® Donkey Anti-Goat IgG (H+L), Jackson Immunoresearch, cat. no: 705-585-147, lot: 145265 IF 1:200;
Alexa Fluor® 488 AffiniPure® Donkey Anti-Rabbit IgG (H+L), Jackson Immunoresearch, cat. no: 711-545-152, lot: 150422 IF 1:200;
Alexa Fluor® 594 AffiniPure® Donkey Anti-Rabbit IgG (H+L), Jackson Immunoresearch, cat. no: 711-585-152, lot: 150227 IF 1:200;
Alexa Fluor® 647 AffiniPure® Donkey Anti-Rabbit IgG (H+L), Jackson Immunoresearch, cat. no: 711-605-152, lot: 149049 IF 1:200.

Validation

Genetic knockdowns were used to validate antibodies targeting FGFR1 and NCAM1. Positive control cell and tissue samples were used to validate antibodies used for IHC and immunofluorescence. Primary antibodies were additionally all validated by the manufacturer. List of statements from manufacturer catalog:
Antibody: abcam ab7646, Statement: Western Blot, IP, ICC/IF;
Antibody: abcam ab59194, Statement: Western Blot, IHC-P, ICC/IF;
Antibody: Cell Signaling 4060S, Statement: Application Key: WB-Western Blotting, IP-Immunoprecipitation, IHC-Immunohistochemistry, IF-Immunofluorescence, F-Flow Cytometry ;
Antibody: Cell Signaling 9272S, Statement: Application Key: WB-Western Blotting, IP-Immunoprecipitation, IF-Immunofluorescence, F-Flow Cytometry;
Antibody: Cell Signaling 9102S, Statement: Application Key: WB-Western Blotting, IP-Immunoprecipitation ;

Antibody: Cell Signaling 9101S, Statement: Application Key: WB-Western Blotting, IP-Immunoprecipitation, IF-Immunofluorescence, F-Flow Cytometry;
Antibody: R&D AF2408, Statement: Applications: Western Blot 0.5 µg/mL, Simple Western 5-25 µg/mL, Flow Cytometry 0.25 µg/10^6 cells, CyTOF-ready;
"Antibody: Cell Signaling 97166S, Statement: Application: Western Blotting 1:1000.
Antibody: Sigma T5168, Statement: Monoclonal Anti-α-Tubulin antibody produced in mouse has been used: In immunofluorescence Analysis, In western blotting/ Immunoblotting, For immunolabelling cells in electron microscopy;
Antibody: Invitrogen PA5-25979, Statement: Applications: Western Blot (WB) 1:1,000, Immunohistochemistry (IHC), Immunohistochemistry (Paraffin) (IHC (P)) 1:10-1:50, Flow Cytometry (Flow) 1:10-1:50;
Antibody: Invitrogen 13-0300, Statement: Applications: Western Blot (WB) :1,000, Immunohistochemistry (IHC) 10-50 µg/mL, Immunohistochemistry (Paraffin) (IHC (P)), Immunohistochemistry (Frozen) (IHC (F)), Immunohistochemistry - Free Floating (IHC (Free)), Immunocytochemistry (ICC/IF);
Antibody: Novusnio NBP3-15367, Statement: Applications: WB, ELISA, ICC/IF, IHC;
Antibody: DAKO M0821, Statement: Optimized for immunohistochemistry (IHC) with validated protocols;
Antibody: Synaptic Systems 106004, Statement: Applications WB: 1 : 1000 (AP staining), ICC: 1 : 1000, IHC: 1 : 500, IHC-P: 1 : 500 up to 1 : 1000;
Antibody: Invitrogen MA1-045, Statement: MA1-045 has been successfully used in immunofluorescence analysis of PSD95 in human motor neurons derived from iPSCs.;
Antibody: Wako 019-19741, Statement: Application : ICC, IHC(Frozen);

Eukaryotic cell lines

Policy information about cell lines and Sex and Gender in Research

Cell line source(s)	Human breast cancer cells UCD12, UCD4, and UCD65 were generated by authors and previously described (PMID: 32576280). Human breast cancer cells MCF7 were purchased from ATCC. Human neuroblastoma cells SH-SY5Y were purchased from the Cell Technologies Shared Resource (RRID: SCR_021982). Human astrocytes (Cat. #1800) were purchased from ScienceCell Research Laboratories. Murine primary astrocytes and neurons were isolated from p0-2 CD1 pups.
Authentication	Cell lines were authenticated by morphology, doubling time, and STR profiling.
Mycoplasma contamination	Cell lines were negative for contamination and tested quarterly.
Commonly misidentified lines (See ICLAC register)	No commonly misidentified cell lines were used in these studies.

Animals and other research organisms

Policy information about studies involving animals; ARRIVE guidelines recommended for reporting animal research, and Sex and Gender in Research

Laboratory animals	NOD.Cg-Prkdc(scid) Il2rg(tm1Wjl)/SzJ (NSG) mice were originally purchased from Jackson Laboratory then bred on-site and injected for in vivo experiments at 8-14 weeks or 52-65 weeks. CD1 breeders were purchased from the Jackson Laboratory. All mice were housed in ventilated cages (maximum 5 mice per cage) with the following conditions:<br>Light cycle: 14 light: 10 dark<br>Temperature: 72°F +/- 2°F<br>Humidity: 40% +/- 10%<br>Water: Hyperchlorinated (2-5 ppm) Reverse Osmosis delivered via automatic watering<br>Food: Teklad (Envigo) diets (2920X)
Wild animals	No wild animals were used.
Reporting on sex	Female animals were used for all studies. Primary cells were derived from male and female mice.
Field-collected samples	No field-collected samples were used.
Ethics oversight	All experiments were approved by the DOD Animal Care and Use Review Office and the Institutional Animal Care and Use Committee (IACUC) of the University of Colorado in an AAALAC accredited, PHS assured, and USDA licensed facility.

Note that full information on the approval of the study protocol must also be provided in the manuscript.

Plants

Seed stocks

Report on the source of all seed stocks or other plant material used. If applicable, state the seed stock centre and catalogue number. If plant specimens were collected from the field, describe the collection location, date and sampling procedures.

Novel plant genotypes

Describe the methods by which all novel plant genotypes were produced. This includes those generated by transgenic approaches, gene editing, chemical/radiation-based mutagenesis and hybridization. For transgenic lines, describe the transformation method, the number of independent lines analyzed and the generation upon which experiments were performed. For gene-edited lines, describe the editor used, the endogenous sequence targeted for editing, the targeting guide RNA sequence (if applicable) and how the editor was applied.

Authentication

Describe any authentication procedures for each seed stock used or novel genotype generated. Describe any experiments used to assess the effect of a mutation and, where applicable, how potential secondary effects (e.g. second site T-DNA insertions, mosiacism, off-target gene editing) were examined.

Magnetic resonance imaging

Experimental design

Design type: Structural MRI for 3D volumetric analysis

Design specifications: Single timepoint

Behavioral performance measures: Not Used

Acquisition

Imaging type(s): Structural high-resolution brain MRI

Field strength: 9.4 Tesla/ 400 MHz

Sequence & imaging parameters: Tri-pilot localizer; Fast spin echo T2-weighted MRI (Bruker TurboRARE: Rapid Acquisition with Relaxation Enhancement) with TR (Repetition time): 2500 ms; TE (Echo time): 33 ms; RARE factor: 8; FOV (Field of view): 20x20 mm; Matrix size: 256x256; Voxel size: 78x78x700 um3

Area of acquisition: Whole brain

Diffusion MRI ☐ Used ☒ Not used

Preprocessing

Preprocessing software: Images were converted to NIFTI format using Bruker2nifti

Normalization: No spatial normalization; Global intensity normalization was achieved by scaling mean image intensities to match across animals

Normalization template: scaling mean image intensities to match across animals

Noise and artifact removal: Not applicable

Volume censoring: Not applied

Statistical modeling & inference

Model type and settings: Not applicable

Effect(s) tested: Not applicable

Specify type of analysis: ☐ Whole brain ☒ ROI-based ☐ Both

Anatomical location(s): Metastases were identified based on visual inspection to ensure accurate ROI placement.

Statistic type for inference (See Eklund et al. 2016): Not applicable

Correction: Not applicable

Models & analysis

n/a	Involved in the study
☒	☐ Functional and/or effective connectivity
☒	☐ Graph analysis
☒	☐ Multivariate modeling or predictive analysis
