# Supplementary material for: Brain FGF2 and NCAM1 contribute to FGFR1-dependent progression of estrogen receptor-positive breast cancer brain metastases
Source: Nat Commun. 2026 May 28;17:6945. doi: 10.1038/s41467-026-73726-5 (PMC13388965; doi:10.1038/s41467-026-73726-5)
Supplement: Supplementary file 9 — Transparent Peer Review file [file 41467_2026_73726_MOESM9_ESM.pdf]

nature portfolio

Peer Review File

Brain FGF2 and NCAM1 contribute to FGFR1-dependent progression of estrogen receptor-positive breast cancer brain metastases.

Corresponding Author: Dr Diana Cittelly

This file contains all reviewer reports in order by version, followed by all author rebuttals in order by version.

Version 0:

Reviewer comments:

Reviewer #1

(Remarks to the Author)
In this manuscript, Dr. Fox and coworker report that FGFR1-amplification, a well-31 established driver of estrogen receptor positive (ER+) breast cancer endocrine resistance, promotes ER+ breast cancer brain metastasis (BCBM) colonization in young and aged mice, through brain-dependent mechanisms. FGFR1-dependent brain colonization in young and aged mice occurs via canonical FGF2/FGFR1 signaling and non-canonical NCAM1/FGFR1 interactions. Astrocytic FGF2-mediated paracrine activation of FGFR1 promoted BCBMs in estrogen-treated young mice, but FGF2 signaling decreased in the brain with aging and estrogen-depletion. Neuronal and glial NCAM1, which remain unchanged in young and aged brains, promoted adhesion to neurons and migration of ER+ breast cancer cells, suggesting that interactions with astrocytes and neurons facilitate early ER+ BCBM colonization through FGFR1. Importantly, FDA-approved FGFR inhibitors blocked early but not late metastatic progression only in young mice, suggesting limited efficacy of FGFR inhibitors to block non-kinase-dependent FGFR1 functions in vivo.

Overall, this is a very good study from an excellent group of investigators and there is novelty in the reported findings. The data are neatly presented; however, they are quite dense and not always easy to follow. Although it might be clear that NCAM is mediating its effects on FGFR signaling via noncanonical mechanisms, it is not clear what those are. In addition to that, there are a number of weaknesses and questions listed below that limit my overall enthusiasm.

Critique

Can the authors clarify if the E2 treated mice are dying sooner from extracranial metastases, thus not having time to develop BCBMs. Are the results affected by lead time bias?

Fig. 1k dot plot does not have statistical analysis. Is there no clear difference in number of metastases?

Fig. 1l is not clear. It would benefit for better contrast.

In line 146, The author stated that “expression of cognate FGFR1-activating ligands (Supplementary Fig.2)”. However, there are no data showing FGFR1-activating ligands expression in Supplementary Fig. 2.

Fig, 2b: It is not clear if the FGFR1 copy number is corrected for centromere 8 signals, which is the rigorous way to assess true gene amplification.

pFGFR1 immunoblot in Fig. 5d is of poor quality. It includes several non-specific bands and is quite different than the one shown in Fig. 2c.

In Figs. 5 and 6, the authors show that FGF2 is the key molecule promoting FGFR1 activation and cancer cell growth. Data would be strengthened by showing the effect of FGF2 using organotypic coculture (as shown in used in Fig.2) with or without a commercially available neutralizing FGF2 antibody. Otherwise, the statement in the Results section “Therefore, these results suggest that FGF2 promotes broad transcriptional changes, driving canonical signaling through FGFR1 and

proliferation, while NCAM1 may drive specific gene signatures promoting neural interactions in ER+ BC cells," has to be modified to acknowledge this limitation.

Two non-selective FGFR1 inhibitors are used in different experiments: ponatinib and BGJ398. Were the results interchangeable and/or consistent with both? Further, 10 micromolar BGJ398 is a highly supra-pharmacological dose which almost certainly has non-FGFR effects.

Fig. 7F: The immunofluorescence images for Synapsin1/2, PSD95, and Puncta appear to be from different tumor regions. The authors should show merged images.

It is quite puzzling that treatment with BGJ398 did not impact BM colonization or extracranial progression in aged mice whereas treatment with the TKI completely suppresses NCAM induced FGFR1 activity (Fig. 8). This begs the unresolved question as to how is NCAM activating FGFR signaling and contributing to BCBMs.

The authors propose that these results may explain why clinical trials have shown that not all patients with ER+ breast cancer receiving non-selective FGFR inhibitors respond to treatment, and why FGFR1 amplification does not predict response to this therapy. The first part of this sentence is not warranted since, to my knowledge, BCBMs were not an endpoint in those studies. Re the 2nd point, the authors should acknowledge that in the majority of FGFR1 amplified tumors, FGFR1 is not the oncogenic driver because of the large size of the 8p amplicon harboring many other genes on which the cancers with high FGFR1 copies are dependent on.

Reviewer #2

(Remarks to the Author)
In the current manuscript, Fox et al. interrogate the role of the tumor microenvironment in FGFR-mediated signaling to promote colonization of the brain and progression of brain metastasis in ER+ breast cancer. While the amplification of FGFR1 has been previously reported to be associated with worse prognosis of ER+ breast cancer, this study provides a more in-depth analysis of the contribution of glial and neuronal compartments to the pro-tumoral signaling of FGFR for ER+ breast cancer brain metastasis. First of all, ER+ breast cancer models that recapitulate the clinical progression of the disease, including its capacity for spontaneous brain colonization, are described. The authors compare the role of FGFR signaling in young and aged ER+ breast cancer brain metastasis models, that express different levels of the ligand FGF2 mainly produced by astrocytes, but comparable levels of NCAM1 derived from both glial and neuronal cells. It is described that FGFR1 promotes brain conization through glial FGF2/ tumor FGFR1 signaling and astrocytic and neuronal NCAM1/FGFR1 axis. They further demonstrate that FGF2-FGFR1 signaling mediates brain metastasis in ER+ breast cancer estrogen-treated young mice, and both neuronal and glial NCAM1-FGFR1 interaction promotes migration of breast cancer brain metastatic tumor cells in young and aged mice. Interestingly, the authors show that FGFR1 promotes increased synaptic puncta with neurons and NCAM1/FGFR1 communication drives interactions between neurons and breast cancer brain metatasis cells.
Finally, they report that activation of FGFR1 by the microenvironment is required for early but not for late stages of brain metastasis and that, FDA-approved FGFR inhibitors only impair brain metastatic progression in young mice and at early stages.
While these findings contribute to a more in deep understanding of the mechanisms driving the understudied ER+ breast cancer brain metastasis, the manuscript suffers from some major issues related to the narrative as well as some additional experiments should be done to ensure that the claims made are supported by experimental data.

Major:
-Regarding brain colonization, ex vivo cultures are not recapitulating BBB crossing and BLI signal in the brain after few hours post-ic injection is not a good indicator of the ability of cancer cells to colonize the brain. Thus, to study the impact of FGFR signaling on the initial stages of brain colonization and validate their conclusions of the the ex vivo and in vivo studies, authors should perform histology for micrometastasis. Additionally, using blood brain barrier studies would discard that impaired FGFR signaling is affecting transmigration before colonizing brain parenchyma.
-As discussed throughout the manuscript, it is not clear the prognostic value of phosphorylation/signaling activation vs total levels of FGFR in either human samples or experimental models.
In vitro experiments are assessing FGFR1 downstream signaling. However, for the in vivo experiments, authors show pFGRF1 expression in established BCBM and disseminated cells few days after ic injection. Number of disseminated cells in young and aged mice showed in Fig. 8b is quantified in the total brain? Performing the downstream signaling in the in vivo context comparing initial vs established and young vs aged mice would help to clarify the functional relevance of the axis in the different contexts.
In this line, authors should discuss the different results of the intracranial effect of the genetic and pharmacological approaches (when applied at initial stages) in aged mice, could it be explained with the role of total and activated FGFR1?
-The authors claim that the functional impact of FGF2/NCAM1-FGFR1 axis is different between initial and late stages of brain metastasis, however experiments in figure 4 show FGF/FGFR signaling in the tumor microenvironment of mice after 6 months of tumor induction. Narrative should be improved including timing considered as initial and established in the experiments depending on the experimental setup and evidence supporting this claim should be shown.
-The immunocompromised mice used for the presented studies should be better described (references suggest athymic nude mice are used for OVX, but NSG mice are used for ic injections and spontaneous dissemination post-OVX). Given the age-related changes in different cell types, including the immune system, alteration in the levels of ligands produced by non-glial cells (authors only focus on GFAP+/Iba1+ area, as well as lysates from isolated mouse and human brain cells -Fig. 4,

Fig. 5 and Extended Data Fig. 4-) could potentially contribute to the extracranial phenotype observed in aged mice but not in young ones and/or different incidence of brain metastasis. Could you exclude the involvement of these cells in the results?
-Regarding the role to the secretion of NCAM1 by different cells types, apart from using neuroblastoma cells, authors should show the effect of depleting NCAM1 from the CM of astrocytes.
-Which is the effect of FGFR inhibition in your cell lines? Authors should discuss if a potential treatment could be used in a preventive scenario for brain metastasis.
-The low incidence of brain metastasis (reflected by ex vivo BLI of the brain and not in vivo BLI) of aged mice in experiments of Fig. 8I, makes difficult to appreciate the lack of phenotype in the treated condition. Is the ex vivo BLI showed in Fig. 8f and Fig. 8I different between controls of young and aged mice? Which is the size and number of the metastasis found in the brain at end point?
Minor:
-The abstract should be revised to better reflect the logical flow and strengthen the connections between the sections of the manuscript.
-Materials and methods section should include the substrate used for BLI assays as the sensitivity varies among different luciferins and this is relevant for the results regarding brain colonization.
-Explain better the use of E2-free media in in vitro experiments.
-The manuscript focusses on the relevance of interactions with astrocytes and neurons from early stages of metastatic colonization, but authors should take into account the different roles of the microenvironment during early and late stages of brain metastasis (Kienast et al. (2010), Valiente et al. (2014), Zhang et al. (2015), Chen et al. (2016), Priego et al. (2018) and others).

Reviewer #3

(Remarks to the Author)
Morgan S. Fox et al developed ER+ breast cancer brain metastasis (BCBM) models by using PDX-derived cell lines in young and aged mice to more accurately capture the progression of ER+ BC in post-menopausal or post-endocrine therapy patients and address how tumor microenvironment contributes to BCBM and resistance to anti-estrogen therapies. Through 3D co-culture assays, in vivo experiments to assess metastatic burden, and spatial transcriptomics, they provide a plausible mechanism by which the current FGFR1 inhibitors are not effective in treating late metastatic progression.

Through experiments involving genetic loss and gain of FGFR1, the authors establish that FGFR1 overexpression is critical for BCBM progression in both young and aged mice. Notably, through spatial profiling of BMs and in vitro studies, they demonstrate that the differential transcriptional profile of surrounding astrocytes rather than the BM cells itself is responsible for promoting BM, wherein it occurs through canonical FGF2/FGFR1 signaling in young mice and non-canonical NCAM1/FGFR1 signaling in aged mice.

Overall, the study provides a clinically relevant ER+ BCBM model, highlights the importance of microenvironment dependent FGFR1 signaling in promoting BCBM and provides a possible reason of why the FGFR1 inhibitors might not function in post-menopausal or post-endocrine therapy patients. However, more mechanistic insights are needed to confirm the functional specificity of FGFR1 functions that play a role in therapy resistance.

To be considered for publication, the following concerns need to be addressed.

• In Figure 2I-2O, the authors utilize co-culture systems to demonstrate that overexpression of FGFR1 in the MCF7 cell line enhances its growth on brain slices. But it is unclear if MCF7-FGFR1-OE cells can metastasize to the brain in vivo, in both young and aged mice. It would be interesting to test this aspect.
• In Figure 2 and Figure 8, the authors show that there is no correlation between FGFR1 and pFGFR1 in patient BM samples as well as in UCD65 cells. In contradiction to this, they utilize pFGFR1 signal as a readout for their in vitro experiments in Figure 5 and Figure 6. Furthermore, in line 147, they conclude 'FGFR1 function may depend on ligand-dependent activation through the TME'. This requires clarification.
• In Figure 6C, based on the pFGFR1 levels, NCAM1 mediated activation of FGFR1 is significantly less compared to that of FGF2. More experiments are required to conclude that NCAM1 indeed activates FGFR1. The authors could also check for other known/novel NCAM1 interactors in metastatic cells.
• To conclude that FGFR1 activity is critical for BCBM, it would be essential to conduct experiments with kinase dead mutants of FGFR1 in young and aged mice.
• In Figure 6, the authors demonstrate that NCAM1 activates FGFR1. Although authors have used BGJ398, it would be worthwhile to conclude this with NCAM1 supplementation in shFGFR1 UCD65 cells.
• In line 271, the authors conclude 'Thus, these results indicate that neuronal and astrocytic NCAM1 can activate FGFR1 in ER+ BC cells upon arrival to the brain niche'. Few points need to be addressed before drawing the conclusion. Although they show NCAM1 FPKM data from brainrnaseq.org (Extended data Figure 4) and utilize astrocyte conditioned media to show FGFR1 activation (Figure 5), there is a lack of direct evidence that astrocytic NCAM1 can activate FGFR1. A similar experiment with astrocyte, like the one shown in Figure 6D-6F, would be essential to directly conclude that astrocytic NCAM1 can activate FGFR1.

Minor points:

• In line 165, the authors mention ''Moreover, FGFR1-KD cells showed decreased brain signal (but not extracranial signal) within 6 hours post-injection in aged mice (Fig. 3f,j)'. Does the authors mean 6 weeks, as 6 hours data is not shown in Figure

3F-3J.

• It would be worthwhile to mention the p-value in Figure 1K and Figure 3C.

Reviewer #4

(Remarks to the Author)
This study investigated FGFR1-dependent ER+ breast cancer brain colonization by exploring the roles of FGFR1 ligands FGF2 and NCAM1 in the tumour microenvironment (TME) during early and late-stage breast cancer brain metastases (BCBM). The authors elegantly utilised aged and estrogen suppressed mouse models more reflective of the TME of patient demographics impacted by ER+ BCBM (postmenopausal women), which has been under-represented in BCBM in vivo studies given most rely on E2 supplementation to drive tumour growth. FGFR1 knockdown tumours showed decreased BCBM progression in young and aged mice showing that FGFR1 is important for early-stage colonisation. Using NanoString GeoMx Digital Spatial Profiling the authors identified that FGF2 was depleted in the aging TME and in estrogen suppression, suggesting alternative ligand interactions may drive FGFR1-dependent BCBM in the aged/E2 suppressed TME. NCAM1 is another FGFR1 ligand and the author’s demonstrated it remained unchanged in the aging TME and provided data that it may facilitate neural interactions with ER+ BC cells driving migration. The authors also showed that early stage BCBM in young mice was more susceptible to FGFR inhibition with BGJ398 then late stage BCBM or in aged mice. This is clinically relevant and supports further exploration for alternative targets in these resistant cohorts. I am supportive of this manuscript progressing if the authors can address the following concerns:

1) The authors propose NCAM1-FGFR1 interactions are important for early-stage BM colonisation in young and old mice. However, FGFR inhibitor BGJ398 showed no impact on early-stage BM colonisation in aged mice (Fig 8.h).
a. Do the authors think this is an issue of dosage as the in vitro assay showed that BGJ398 was able to inhibit NCAM1-induced FGFR1 activation (Fig 6c)? I would suggest the authors explore alternative drug delivery routes to increase BGJ398 concentrations in the brain. It is difficult to draw the conclusion that NCAM1-FGFR1 interaction is important in brain colonisation of aged mice if it is not clear that the interaction is effectively inhibited by BGJ398.
b. Does membrane pFGFR1 detection/intensity decrease following BGJ398 treatment in aged mice during early stage BCBM compared with untreated? This could be an effective readout to ensure the drug is effectively blocking FGFR1 activation.
2) The number of ROIs per condition in the NanoString GeoMx Digital Spatial Profiling assay was outlined but can the authors clarify in the methods how many mice were used per condition?
3) Can the authors also elaborate in the methods on how the GFAP+ and Iba+ glial areas were selected as ROIs? The example image in Fig 4f shows inclusion of glial cells more distal (not in direct contact with the PanCK positive cells) included in the analysis mask – was there a maximum distance of the analysis mask from panCK cells or a maximum ROI area size?
4) Given that the tumour microenvironment (TME) differs between young and old mice, it is reasonable to expect that tumour cells may engage alternative signalling pathways to support growth in E2 depleted TMEs. The authors show via UMAPs (Fig. 4d) that there were no major transcriptomic shifts within the tumour cells (in contrast to the more pronounced TME differences shown in Fig. 4g). However smaller but still relevant changes could still be picked up by differential gene expression analysis between tumour cells from young and aged mice to identify pathway-level differences. This could be relevant for identifying alternative therapeutic targets in late stage BCBM in aged populations given FGFR inhibitors maybe ineffective. If this is beyond the scope of the study, I would suggest to de-emphasise that the tumours were “transcriptionally similar in young and aged mice” as mentioned in the discussion, rather that there were larger global differences in the TME compared with tumour in young and aged mice.
Minor issues:
1) Typo line 348 - BJG398 instead of BGJ398.

Version 1:

Reviewer comments:

Reviewer #1

(Remarks to the Author)
The authors have satisfactorily addressed all my comments.

Reviewer #2

(Remarks to the Author)
The authors have satisfactorily addressed all of my questions and concerns by conducting additional experiments and by revising and clarifying the manuscript accordingly.

Reviewer #3

(Remarks to the Author)
Authors have made sincere attempt to address the concerns raised and the manuscript is much improved. I accept it for

publication.

Reviewer #4

(Remarks to the Author)
The authors have satisfactorily addressed my concerns and significantly strengthened the manuscript through additional mechanistic experiments and analyses. The revised version presents compelling evidence for the roles of FGF2/FGFR1, and non-canonical NCAM1/FGFR1 signalling, in ER+ breast cancer brain metastasis colonisation, as well as how age-related changes in these pathways influence colonisation. I have no further major concerns.

Open Access This Peer Review File is licensed under a Creative Commons Attribution 4.0 International License, which permits use, sharing, adaptation, distribution and reproduction in any medium or format, as long as you give appropriate credit to the original author(s) and the source, provide a link to the Creative Commons license, and indicate if changes were made.
In cases where reviewers are anonymous, credit should be given to 'Anonymous Referee' and the source.
The images or other third party material in this Peer Review File are included in the article's Creative Commons license, unless indicated otherwise in a credit line to the material. If material is not included in the article's Creative Commons license and your intended use is not permitted by statutory regulation or exceeds the permitted use, you will need to obtain permission directly from the copyright holder.
To view a copy of this license, visit https://creativecommons.org/licenses/by/4.0/

We thank the reviewers for their careful review of our manuscript and believe that addressing their concerns with multiple additional studies and clarifications has significantly strengthened the conclusions. Please find below a point-by-point response to all questions.

Reviewer #1

1) *Can the authors clarify if the E2 treated mice are dying sooner from extracranial metastases, thus not having time to develop BCBMs. Are the results affected by lead time bias?*

R: In the spontaneous metastases' models (**Supplementary Fig. 1a-j**), E2-treated mice do die first from primary tumor recurrence and extracranial metastases, which prompted us to use intracardiac injection to study brain colonization in the subsequent studies. When using intracardiac injection, E2-supplementation increases metastatic burden in all organs, however, mice are not dying from extracranial metastases prior to development of BMs, as they are *developing BMs concurrent with extracranial* disease (**Fig. 1f-h**). Metastases (including BMs) develop slowly in aged mice when compared to young E2-treated mice. However, the results in **Fig. 1g-l**, show increased BM in both aged *and* young-E2-treated mice despite all mice being euthanized at the same timepoint.

2) *Fig. 1k dot plot does not have statistical analysis. Is there no clear difference in number of metastases?*

R: Former Fig 1K depicted the macroscopic visualization of large GFP+ metastases through multiple experiments. To address the concern of this and other reviewers, we have replaced this quantification by a more accurate histological quantification of metastases in mice from panels **Fig. 1e-g**, which shows the statistically significant increase in brain metastatic clusters in young/E2-treated mice, as well as aged naïve mice compared to Young OVX mice (now **Fig. 1h**). Lines 103-115.

3) *Fig. 1l is not clear. It would benefit for better contrast.*

R: We have darkened the mask of the tumor regions in MRI images to clearly indicate the BCBM. Unmasked images can still be found in **Supplementary Fig. 1k,l**.

4) *In line 146, The author stated that "expression of cognate FGFR1-activating ligands (Supplementary Fig.2)". However, there are no data showing FGFR1-activating ligands expression in Supplementary Fig. 2.*

R: Our original submission included both Extended Data and Supplementary Figures. While Sup. Fig. 2 contained the referred to data, Extended Data Fig. 2 did not. Based on editorial requirements for Nature Communications, we have consolidated Extended Data Figures and Supplementary Figures. The new **Supplementary Fig. 2a** now contains the table of FGF mRNA expression for UCD46, UCD4, UCD12, and UCD65 cell lines.

5) *Fig, 2b: It is not clear if the FGFR1 copy number is corrected for centromere 8 signals, which is the rigorous way to assess true gene amplification.*

R: Gene copy number alterations for the UCD12 and UCD65 cell lines were reported by the investigators who developed and characterized the lines[1] and were identified by the Oncomine Comprehensive Assay v3 panel (Thermo-Fisher), an NGS platform which detects SNVs, CNVs, gene fusions, and indels from 161 cancer driver genes. This assay, however, does not count centromeres. Despite this limitation, we hope the reviewer agrees the cells provide relevant models to study the role of FGFR1 signaling as they have high levels of FGFR1 mRNA and protein expression.

6) *pFGFR1 immunoblot in Fig. 5d is of poor quality. It includes several non-specific bands and is quite different than the one shown in Fig. 2c.*

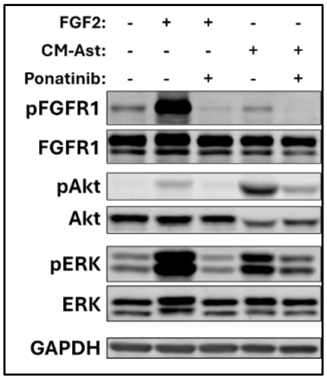

R: We agree with the reviewer that the original immunoblots contained some unspecific bands. While original Fig. 2c and Fig. 5d used the same antibody (Abcam ab59194), we observed some variability in the strength and MW of the bands with this antibody between experiments and had identified a more consistent antibody (Cell Signaling 52928s), used for all experiments in Fig. 6. In response to the reviewer's concern, we repeated the WBs from Fig. 2c and 5d with the CS antibody, detecting consistent pFGFR1 bands. However, this new antibody showed that in former Fig. 5d (see blot on the right), the conditioned media did not induce pFGFR1,

and the blockade of downstream Akt and ERK activation was likely due to the blockage of other survival pathways by the multi-TKI inhibitor Ponatinib. We further confirmed that conditioned media-induced Akt and ERK activation were not FGFR1-dependent, as these could not be blocked with the more specific FGFR inhibitor BGJ398 (now **Supplementary Fig. 5e**). These results prompted us to reinterpret the mechanisms whereby astrocytic FGF2 could impact FGFR1 activation in a paracrine manner. We measured FGF2 protein levels in the CM-mAst using ELISA and found that FGF2 is not secreted in the CM (now **Supplementary Fig. 5c**). Prior studies have shown that FGF2 is primarily membrane-bound[2-4], and we found that FGF2 is expressed in the astrocytic cell surface when in high-density co-culture with cancer cells (now **Supplementary Fig. 5d**). Thus, while astrocytes express FGF2, present it to the cell surface, and activate FGFR1 in cancer cells, this effect is cell-contact dependent and not through the conditioned media as we initially interpreted. We have added this new data (lines 190-198) and modified the conclusions of the manuscript accordingly (lines 328-334).

7) *In Figs. 5 and 6, the authors show that FGF2 is the key molecule promoting FGFR1 activation and cancer cell growth. Data would be strengthened by showing the effect of FGF2 using organotypic coculture (as shown in used in Fig.2) with or without a commercially available neutralizing FGF2 antibody. Otherwise, the statement in the Results section "Therefore, these results suggest that FGF2 promotes broad transcriptional changes, driving canonical signaling through FGFR1 and proliferation, while NCAM1 may drive specific gene signatures promoting neural interactions in ER+ BC cells," has to be modified to acknowledge this limitation.*

R: We thank the reviewer for this suggestion and have performed experiments using an FGF2 neutralizing antibody in organotypic brain slices, that demonstrated blockage of FGF2 decreases UCD12 spheroid growth in organotypic co-culture (**Fig. 5i**). We additionally downregulated FGF2 in primary astrocytes in vitro, and showed that FGF2 KD in astrocytes decreases UCD12 and UCD65 proliferation (now **Fig. 6m,n**). We have modified the results and conclusions accordingly (lines 188-190, 222-229).

8) *Two non-selective FGFR1 inhibitors are used in different experiments: ponatinib and BGJ398. Were the results interchangeable and/or consistent with both? Further, 10 micromolar BGJ398 is a highly supra-pharmacological dose which almost certainly has non-FGFR effects.*

R: The reviewer is correct; our earlier studies used the less selective FGFR1 inhibitor ponatinib. We have compared the specificity of these two inhibitors to block FGF2/FGFR1 (now **Supplementary Fig. 5c**, response to #6 here), and have now removed Ponatinib from that panel, as BGJ398 demonstrated superior specificity. We have additionally repeated signaling immunoblots with lower doses of BGJ398 and found that 10-fold and 100-fold lower BGJ398 concentrations effectively block FGF2 and NCAM1-induced FGFR1 and downstream activation (**Supplementary Fig. 6e**).

9) *Fig. 7F: The immunofluorescence images for Synapsin1/2, PSD95, and Puncta appear to be from different tumor regions. The authors should show merged images.*

R: The IF images are split channels from the same areas marked by the yellow box. Since puncta are defined by proximity of PSD95 and Synapsin, the puncta do not always represent a full overlap of these two markers[5]. We have now added a merged panel for Synapsin1/2, PSD95 and puncta to better visualize this.

10) *It is quite puzzling that treatment with BGJ398 did not impact BM colonization or extracranial progression in aged mice whereas treatment with the TKI completely suppresses NCAM induced FGFR1 activity (Fig. 8). This begs the unresolved question as to how is NCAM activating FGFR signaling and contributing to BCBMs.*

R: We agree that the lack of effect from the TKI in aged mice was unexpected and warranted further investigation. A blocking ab against NCAM1 showed that NCAM1 depletion decreases the growth of ER+ BC cells in the aged brain but not in the young brain (now **Fig. 6k,l**), and that astrocytic NCAM1 activates pFGFR1 and drives growth of BC cells (now **Fig. 6i,m,n**), supporting the notion that NCAM1 can drive FGFR1+ cell growth in the absence of FGF2. The "intensity" of pFGFR1 is lower in the aged brain compared to young hosts (**Fig. 8a**), likely reflecting the higher affinity and ability of FGF2 to induce FGFR1 activation. This is consistent with the slower progression of metastatic burden in aged mice compared to E2-treated young mice (compare IVIS signal in Fig. 8f to 8k), which could contribute to the inability of IVIS to detect significant changes in ex vivo brains. Unfortunately, these studies were terminated due to the high toxicity of the FGFR inhibitor in aged mice, which precluded further growth of metastases. Nonetheless, per the suggestion of Reviewer 2, we completed histological quantification of metastases in all our studies and found that BGJ398 treatment from the

time of cell injection did reduce the total number of intraparenchymal BM clusters in aged mice at euthanasia (now **Fig. 8l**). We have incorporated these new data and modified the interpretation of the results accordingly (lines 277-298)

11) *The authors propose that these results may explain why clinical trials have shown that not all patients with ER+ breast cancer receiving non-selective FGFR inhibitors respond to treatment, and why FGFR1 amplification does not predict response to this therapy. The first part of this sentence is not warranted since, to my knowledge, BCBMs were not an endpoint in those studies. Re the 2nd point, the authors should acknowledge that in the majority of FGFR1 amplified tumors, FGFR1 is not the oncogenic driver because of the large size of the 8p amplicon harboring many other genes on which the cancers with high FGFR1 copies are dependent on.*

R: We have acknowledged the tumorigenic potential of FGFRamp may result from other 8p co-amplified genes serving as oncogenic drivers (lines 317-322). While FGFR1 may not be the main oncogenic driver of these tumors, our mechanistic studies support the notion that paracrine activation influences FGFR1 function, particularly in early stages of BMs and could influence the window of opportunity for the use of FGFR1 inhibitors in BMs.

Reviewer #2

1) *Regarding brain colonization, ex vivo cultures are not recapitulating BBB crossing and BLI signal in the brain after few hours post-ic injection is not a good indicator of the ability of cancer cells to colonize the brain. Thus, to study the impact of FGFR signaling on the initial stages of brain colonization and validate their conclusions of the the ex vivo and in vivo studies, authors should perform histology for micrometastasis.*

R: We agree with the reviewer, *ex vivo* culture does not recapitulate BBB crossing and our studies do not attempt to elucidate the impact of FGFR1 in extravasation, but rather the survival of disseminated cells and their outgrowth into large metastases. Our initial description of BLI signal 6hr after injection was confusing as we were not attempting to measure extravasation, so we have removed this from the manuscript. To further address the reviewer's concern, we have performed histological quantification of brain metastasis for all *in vivo* experiments where tissue was collected (**Fig. 1h,l; Fig. 3c,g,k; Fig. 8g,l**). We further demonstrate that *in vivo* head BLI signal at endpoint correlates with clusters of brain metastases identified histologically (**Supplementary Fig. 1m**). We use a previously described method for metastatic quantification that defines micrometastases (<300um) and metastatic clusters (micro and macrometastases >300um in defined regional areas)[6-8]. While we did not perform histological quantification in any animals euthanized at early timepoints (mostly due to the limited availably of aged NSG mice-which are not available commercially), we have shown similar numbers of disseminated cells in the brain parenchyma in young and aged animals through IF (now **Fig. 8b**).

2) *As discussed throughout the manuscript, it is not clear the prognostic value of phosphorylation/signaling activation vs total levels of FGFR in either human samples or experimental models. In vitro experiments are assessing FGFR1 downstream signaling. However, for the in vivo experiments, authors show pFGRF1 expression in established BCBM and disseminated cells few days after ic injection. Number of disseminated cells in young and aged mice showed in Fig. 8b is quantified in the total brain? Performing the downstream signaling in the in vivo context comparing initial vs established and young vs aged mice would help to clarify the functional relevance of the axis in the different contexts.*
In this line, authors should discuss the different results of the intracranial effect of the genetic and pharmacological approaches (when applied at initial stages) in aged mice, could it be explained with the role of total and activated FGFR1?

R: We have revised the manuscript to better reflect that:1) total FGFR1 status does not correlate with pFGFR1 levels, and that overexpression does not lead to autophosphorylation but makes cells susceptible to activation by the TME, and 2) TME-dependent activation of pFGFR1 impacts early but not late stages of BM progression. In Fig. 8b (now **Fig. 8a**), disseminated cells in the brain were quantified in three 40µm sections of tissue 300µm apart, we have clarified this in the methods. We also emphasize that the differences in the intensity of pFGFR1 staining in young vs aged mice (**Fig. 8a**) are consistent with a strong FGF2-driven activation in younger mice, and a weaker NCAM-driven activation in older mice. We performed quantification of pFGFR1 in

disseminated cells in aged mice 5 days post-injection and show that pharmacological inhibition (BJG398) can penetrate the brain and reduce pFGFR1 levels at this early time point in aged mice (now **Supplementary Fig. 8a**). Thus, it is likely that the differences in genetic (FGFR1 KD) vs pharmacologic response (BJG398) for decreased brain colonization result from the slower progression of BM in aged mice and the major toxicities of BGJ398 in aged mice, which prevented longer BM progression. Our histological quantification (in response to this reviewer's suggestion) now revealed a decrease in intraparenchymal metastases in aged mice treated with BGJ398 since the day of injection that we had not observed via IVIS, suggesting there was activity of the inhibitor even in aged mice (please see more details below in response to Q7). While we appreciate the reviewer's suggestion to use IF to parse out specific downstream signaling in all stages of BM in vivo, we had limited availability of aged mice to assess early stages of dissemination. In addition to verifying p-FGFR1 levels in disseminated cells in young and aged mice, we performed IF in late-stage BM from young mice treated with vehicle or BGJ398 and observed similar levels of pERK in BMs from vehicle vs BGJ398 treated mice (W0-12) in young mice (**Supplementary Fig. 8d**). This is consistent with our conclusion of FGFR-signaling independence at late-stages of BMs, as well as our data showing that conditioned media from astrocytes induce activation of Akt and ERK in cancer cells through mechanisms that don't rely on FGFR1 (**Supplementary Fig. 5e).** It is possible that some of these mechanisms are in part responsible for the FGFR1-independent progression of late-stage brain metastases that render them insensitive to FGFR1 inhibition. We have added these data and revised conclusions to the manuscript (lines 262-298).

3) *The authors claim that the functional impact of FGF2/NCAM1-FGFR1 axis is different between initial and late stages of brain metastasis, however experiments in figure 4 show FGF/FGFR signaling in the tumor microenvironment of mice after 6 months of tumor induction. Narrative should be improved including timing considered as initial and established in the experiments depending on the experimental setup and evidence supporting this claim should be shown.*

R: While Nanostring experiments to elucidate changes in the glial or cancer cells in Fig. 4 were performed in animals harboring late-stage BCBMs (6 months post injection), we confirmed the levels of FGF2 decreased in young vs aged mice in tumor-naïve mice (**Fig. 5a**), and others have reported decreases of brain FGF2 following E2-suppresion[9]. We hope that additional data addressing the roles of FGF2 vs NCAM in young and old organotypic slices, and KDs of FGF2 and NCAM1 in astrocytes (in response to Q5, below, and to Q7, reviewer 1), provides further evidence of the two axes used by FGFR1 to colonize the brain in young and aged mice. The revised manuscript has better explained the dual mechanisms leading to FGFR1 activation in the brain considering the changes on FGF2 levels with aging and E2 suppression.

4) *The immunocompromised mice used for the presented studies should be better described (references suggest athymic nude mice are used for OVX, but NSG mice are used for ic injections and spontaneous dissemination post-OVX). Given the age-related changes in different cell types, including the immune system, alteration in the levels of ligands produced by non-glial cells (authors only focus on GFAP+/Iba1+ area, as well as lysates from isolated mouse and human brain cells -Fig. 4, Fig. 5 and Extended Data Fig. 4-) could potentially contribute to the extracranial phenotype observed in aged mice but not in young ones and/or different incidence of brain metastasis. Could you exclude the involvement of these cells in the results?*

R: We have further clarified in the methods that all *in vivo* studies were completed in NSG mice. The reference cited using nude mice (Osborne *et al*) attempted to highlight the dependency of ER+ BC models on E2-supplementation *in vivo*. We and others have shown that this dependency is recapitulated in NSG mice[10,11] (**Fig. 1e-h**).

We agree with the reviewer that we cannot exclude age-related changes in other cell types or their contribution to BMs. We focused on glial cells due to known function of astrocytes as key modulators of survival and growth of disseminated cancer cells, as well as major sources of FGF2 in the brain. While the effects of aging on the immune system and increased relative fitness of ER+ BC cancers warrant further investigation, there are no immunocompetent mouse models of ER+ BC that mirror the acquisition of FGFR1 amplification and endocrine responsiveness in vivo.

Regarding the extracranial effects of FGFR1 on growth in aged mice, prior work has demonstrated that FGFR1 is important in primary tumor growth for obese postmenopausal mice and for bone metastasis growth, both through TME-dependent factors[12,13]. Thus, other mechanisms are likely to contribute to FGFR1-dependent extracranial growth in aged mice. A key significant finding in our studies, however, is that in young/E2 supplemented mice metastases are dependent on FGFR1 in the brain but not extracranially, further

highlighting the relevance of non-E2 dependent mechanisms for brain colonization. We have added this to the discussion (Lines 309 to 322).

5) *Regarding the role to the secretion of NCAM1 by different cells types, apart from using neuroblastoma cells, authors should show the effect of depleting NCAM1 from the CM of astrocytes.*

R: To address the reviewer suggestions, we used NCAM1 KD in primary mAsts and showed that similar to the SH-SY5Y cells, downregulation of astrocytic NCAM1 results in decreased pFGFR1 staining in UCD12 and UCD65 cells (now **Fig. 6i,j**). We also demonstrated that FGFR1+ BC cells co-cultured with NCAM1 KD astrocytes showed decreased growth as compared to cells co-cultured with shNC astrocytes (now **Fig. 6m,n**). We hope that this, and other requested studies (see response to reviewer 1, 3), better support the conclusions regarding the role of NCAM1 to FGFR1 activation and function.

6) *Which is the effect of FGFR inhibition in your cell lines? Authors should discuss if a potential treatment could be used in a preventive scenario for brain metastasis.*

R: We have added data demonstrating that BGJ398 decreased the proliferation of cancer cells in response to FGF2 *in vitro* (**Supplementary Fig. 5f**). We agree with the reviewer that our in vivo data supports FGFR1 inhibition in the preventative, but not therapeutic setting for ER+ BCBM and have added this to the discussion.

7) *The low incidence of brain metastasis (reflected by ex vivo BLI of the brain and not in vivo BLI) of aged mice in experiments of Fig. 8I, makes difficult to appreciate the lack of phenotype in the treated condition. Is the ex vivo BLI showed in Fig. 8f and Fig. 8I different between controls of young and aged mice? Which is the size and number of the metastasis found in the brain at end point?*

R: The reviewer is correct that young animals develop larger metastases (and therefore very high BLI readings, **Fig. 1e-h**), as these mice were treated with premenopausal levels of E2, and these cells remain E2-responsive. By contrast, metastases in older mice are smaller and slow growing, which is reflected in lower the *ex vivo* IVIS signal. We also noted in the revised manuscript that this study had to be terminated due to the significant toxicity of BJG398 in aged mice, which precluded the development of larger metastases. To address the reviewer's concern, we performed histological quantification of micrometastases (<300um), and metastatic clusters (micro and macrometastases) for all groups (**Fig. 8g,l**), which revealed a previously undetected effect of BGJ398 to decrease BM clusters and micrometastases in aged animals. We have modified our conclusions accordingly.

Minor:

8) *The abstract should be revised to better reflect the logical flow and strengthen the connections between the sections of the manuscript.*

R: We have made an effort to improve the clarity of the abstract within the allotted space for publication.

9) *Materials and methods section should include the substrate used for BLI assays as the sensitivity varies among different luciferins and this is relevant for the results regarding brain colonization.*

R: We have added this information to the methods.

10) *Explain better the use of E2-free media in in vitro experiments.*

R: We have added this information in all figure legends, specifying the use of estrogen-free (charcoal-stripped FBS, no phenol-red) versus starvation (serum-free, no phenol-red) media.

11) *The manuscript focusses on the relevance of interactions with astrocytes and neurons from early stages of metastatic colonization, but authors should take into account the different roles of the microenvironment during early and late stages of brain metastasis (Kienast et al. (2010), Valiente et al. (2014), Zhang et al. (2015), Chen et al. (2016), Priego et al. (2018) and others).*

R: We agree with the reviewer that we focused on the relevance of astrocytes and neurons and acknowledge the complexity of the brain and the contribution of multiple cell lines to early and late stages of brain metastases (lines 323-334). We have added some of these key references as suggested.

Reviewer #3

1) *In Figure 2I-2O, the authors utilize co-culture systems to demonstrate that overexpression of FGFR1 in the MCF7 cell line enhances its growth on brain slices. But it is unclear if MCF7-FGFR1-OE cells can metastasize to the brain in vivo, in both young and aged mice. It would be interesting to test this aspect.*

R: The reviewer brings up an important point. As previously reported, the MCF7 cell line is unable to form tumors in the absence of E2 supplementation and are generally poorly metastatic *in vivo*. To address the reviewer'S concern, we injected MCF7 control vs FGFR1-OE cells in young mice supplemented with E2 and found that FGFR1-OE increases BCBM frequency (**Supplementary Fig. 3a-d**). In aged mice without E2 supplementation, very few animals supported growth of these cells upon intracarotid injection (just 1/7 mice developed tumors in the FGFR1-OE group compared to 0/9 in the EV control), suggesting that while FGFR1-OE may increase the frequency of colonization, it is not sufficient to overcome the dependency on E2 for tumor growth *in vivo* (**Supplementary Fig. 3e,f**).

2) *In Figure 2 and Figure 8, the authors show that there is no correlation between FGFR1 and pFGFR1 in patient BM samples as well as in UCD65 cells. In contradiction to this, they utilize pFGFR1 signal as a readout for their in vitro experiments in Figure 5 and Figure 6. Furthermore, in line 147, they conclude 'FGFR1 function may depend on ligand-dependent activation through the TME'. This requires clarification.*

R: We apologize for causing this confusion. The purpose of showing the lack of correlation between total and pFGFR1 in patient samples was to demonstrate that high levels of FGFR1 (and potentially the pro-metastatic effects of FGFR1 amplification) cannot be explained by autophosphorylation (as it can occur with many tyrosine kinase receptors where abundance triggers activation, line 126). We also show this is true in FGFR1 amplified cell lines, and that interactions with the TME (Fig. 5, 6) lead to FGFR1 activation and proliferative signaling in cancer cells. Our novel data also shows that TME-mediated activation of FGFR1 is particularly important at early stages of metastatic colonization, but dispensable at late-stages, as the tumors are no longer responsive to FGFR inhibition. The lack of correlation between total FGFR1 and pFGFR1 in clinical samples (which are all late-stage BMs), is consistent with the FGFR1 independence at these late stages (lines 279-291).

3) *In Figure 6C, based on the pFGFR1 levels, NCAM1 mediated activation of FGFR1 is significantly less compared to that of FGF2. More experiments are required to conclude that NCAM1 indeed activates FGFR1. The authors could also check for other known/novel NCAM1 interactors in metastatic cells.*

R: The reviewer is correct that NCAM1 binds less strongly to FGFR1 compared to FGF2, and our data supports the notion that pFGFR1 levels are stronger in younger mice (with high FGF2) compared to aged mice (where we propose NCAM1 is the main activator). Due to limitations in *in vitro* solubility of recombinant NCAM1 protein, we utilized ~250nM NCAM1 for in vitro studies; however, concentrations on the cell surface are reported to be higher. Our original manuscript had used co-culture experiments with NCAM1 KD in SY5Y cells vs control cells to demonstrate direct FGFR1 activation from NCAM1 on the cell surface. In response to the reviewer concerns, we have now downregulated NCAM1 in astrocytes and showed that cancer cells have decreased pFGFR1 in co-culture with NCAM1-KD astrocytes compared to EV astrocytes (**Fig. 6i,j**). Moreover, we have shown that ER+ BC cells have impaired proliferation when co-cultured with shNCAM1 vs control astrocytes (**Fig. 6m,n**), providing further evidence that NCAM1-driven activity in ER+ BC cells can impact BM growth. Furthermore, using a blocking antibody against NCAM1 decreases proliferation of UCD12 cells when co-cultured with organotypic brain slices from aged mice (with low FGF2), but not in slices from young mice where FGF2 is likely to be the main driver (Now **Fig. 6k,l**).

4) *To conclude that FGFR1 activity is critical for BCBM, it would be essential to conduct experiments with kinase dead mutants of FGFR1 in young and aged mice.*

R: We agree that experiments using kinase-dead FGFR1 mutants would provide valuable additional mechanistic insight into the role of FGFR1 in BCBMs. However, the generation, validation and in vivo characterization of such models in young and aged mice, would require substantial development, and access to >52-week-old NSG mice (that are not commercially available) and thus are beyond the scope of the current study. Notably, our pharmacological inhibition experiments, that now include histological quantification of micro and macrometastases, demonstrate that blocking FGFR1 kinase activity does reduce brain colonization in young and aged hosts.

5) *In Figure 6, the authors demonstrate that NCAM1 activates FGFR1. Although authors have used BGJ398, it would be worthwhile to conclude this with NCAM1 supplementation in shFGFR1 UCD65 cells.*

R: We have completed this experiment (**Fig. 6b**), demonstrating the shFGFR1 UCD65 cells have decreased FGFR1 and downstream signaling activation following FGF2 and NCAM1 stimulation.

6) *In line 271, the authors conclude 'Thus, these results indicate that neuronal and astrocytic NCAM1 can activate FGFR1 in ER+ BC cells upon arrival to the brain niche'. Few points need to be addressed before drawing the conclusion. Although they show NCAM1 FPKM data from brainrnaseq.org (Extended data Figure 4) and utilize astrocyte conditioned media to show FGFR1 activation (Figure 5), there is a lack of direct evidence that astrocytic NCAM1 can activate FGFR1. A similar experiment with astrocyte, like the one shown in Figure 6D-6F, would be essential to directly conclude that astrocytic NCAM1 can activate FGFR1.*

R: We agree with the reviewer that these experiments were critical. We have now demonstrated that NCAM1 KD in mAsts reduced UCD65 and UCD12 pFGFR1 staining in co-culture, and decreased proliferation of ER+ BC cells. We also showed that a blocking antibody against NCAM1 decreases the proliferation of UCD12 in organotypic slices from aged but young brains, which further supports the conclusion that NCAM1 can activate FGFR1 and drive growth in BMs, particularly in the aged brain with low FGF2 levels (**Fig. 6i-n**).

Minor points:

7) *In line 165, the authors mention "Moreover, FGFR1-KD cells showed decreased brain signal (but not extracranial signal) within 6 hours post-injection in aged mice (Fig. 3f,j)'. Does the authors mean 6 weeks, as 6 hours data is not shown in Figure 3F-3J.*

R: We have added clarification that this refers to week 0 IVIS data (which are acquired within 6 hours of cell injection), and changed the text to remove the confusing language.

8) *It would be worthwhile to mention the p-value in Figure 1K and Figure 3C.*

R: Former Fig. 1K depicted the macroscopic visualization of large GFP+ metastases through multiple experiments, with the intent to demonstrate the existence of large true brain metastases, but was not designed to be tested statistically (as the endpoints of some of the experiments differed between samples). We have replaced the GFP+ macrometastasis quantification with the more rigorous histological metastasis quantification, including statistical analysis. In **Fig. 3c**, we have similarly replaced the head IVIS signal quantification at endpoint with histological metastasis quantification, including statistical analysis.

Reviewer #4

1) The authors propose NCAM1-FGFR1 interactions are important for early-stage BM colonisation in young and old mice. However, FGFR inhibitor BGJ398 showed no impact on early-stage BM colonisation in aged mice (Fig 8.h). Do the authors think this is an issue of dosage as the in vitro assay showed that BGJ398 was able to inhibit NCAM1-induced FGFR1 activation (Fig 6c)? I would suggest the authors explore alternative drug delivery routes to increase BGJ398 concentrations in the brain. It is difficult to draw the conclusion that NCAM1-FGFR1 interaction is important in brain colonisation of aged mice if it is not clear that the interaction is effectively inhibited by BGJ398.

Does membrane pFGFR1 detection/intensity decrease following BGJ398 treatment in aged mice during early stage BCBM compared with untreated? This could be an effective readout to ensure the drug is effectively blocking FGFR1 activation.

R: We thank the reviewer for this suggestion. We have performed experiments (Now **Supplementary Fig. 8a**) showing that at the used dose, BGJ398 penetrates the brain and is able to decrease pFGFR1 in disseminated cancer cells in aged mice; thus, the differences in response do not appear linked to drug penetrance. Our new data shows that NCAM1 depletion does impact the growth of cancer cells in organotypic slices in aged but not in young brain (**Fig. 6k,l**), and that BGJ398 caused a significant decrease in the number of intraparenchymal micrometastases and metastatic clusters in aged mice (now **Fig. 8l**), despite no differences in ex vivo IVIS levels. We have now emphasized that metastases do develop slowly in aged mice (which are not E2 supplemented as in young mice) and BGJ398 caused severe toxicities in aged mice, which demanded

termination of the experiment before metastases reached larger sizes (i.e more sensitive to IVIS detection). We believe the histological quantification provides evidence that the early blockage of FGFR1 with BGJ398 impairs brain colonization, even in these aged mice with slower tumor progression.

2) The number of ROIs per condition in the NanoString GeoMx Digital Spatial Profiling assay was outlined but can the authors clarify in the methods how many mice were used per condition?

R: We have added this to the methods. Due to cost constraints, only a single slide was used for NanoString DSP for each species library. We included 1 coronal section of brain-tumor containing tissue from a single mouse for each condition, with each section containing multiple micro- and macrometastases, which were used to define the analysis ROIs. Recognizing this limitation in power, we validated that the decreases in FGF2 observed in glial cells occurred at the protein level in tumor naïve mice.

3) Can the authors also elaborate in the methods on how the GFAP+ and Iba+ glial areas were selected as ROIs? The example image in Fig 4f shows inclusion of glial cells more distal (not in direct contact with the PanCK positive cells) included in the analysis mask – was there a maximum distance of the analysis mask from panCK cells or a maximum ROI area size?

R: We have added this information to the methods. We used a standard field of view for all murine analysis ROIs surrounding panCK+ BMs, and all glial cells within this field were included in the analysis. The maximum possible distance from a cancer cell to a glial ROI was ~400μm.

4) Given that the tumour microenvironment (TME) differs between young and old mice, it is reasonable to expect that tumour cells may engage alternative signalling pathways to support growth in E2 depleted TMEs. The authors show via UMAPs (Fig. 4d) that there were no major transcriptomic shifts within the tumour cells (in contrast to the more pronounced TME differences shown in Fig. 4g). However smaller but still relevant changes could still be picked up by differential gene expression analysis between tumour cells from young and aged mice to identify pathway-level differences. This could be relevant for identifying alternative therapeutic targets in late stage BCBM in aged populations given FGFR inhibitors maybe ineffective. If this is beyond the scope of the study, I would suggest to de-emphasise that the tumours were "transcriptionally similar in young and aged mice" as mentioned in the discussion, rather that there were larger global differences in the TME compared with tumour in young and aged mice.

R: We agree with the reviewer and changed the language to reflect this. We also have added the pairwise differential gene expression analysis information for the human ROIs. There were only 6 differentially expressed genes and no enriched or repressed gene signatures via GSEA (**Fig. 4e**). To the reviewers point about tumor cells engaging in alternative pathways, the fact that the FGFR1 inhibitor is not effective in late stage BM (in either young or aged mice), added to the minimal changes in gene expression among tumors, highlight the fact that astrocytes (and likely other cells in the brain) secrete additional growth factors that can induce pro-survival signaling in cancer cells (in and FGFR1-independent manner).

5) Minor issues: Typo line 348 - BJG398 instead of BGJ398.

R: This has been fixed.

References

1 Finlay-Schultz, J. *et al.* New generation breast cancer cell lines developed from patient-derived xenografts. *Breast Cancer Res* **22**, 68 (2020). https://doi.org/10.1186/s13058-020-01300-y
2 Beenken, A. & Mohammadi, M. The FGF family: biology, pathophysiology and therapy. *Nat Rev Drug Discov* **8**, 235-253 (2009). https://doi.org/10.1038/nrd2792
3 Plotnikov, A. N., Schlessinger, J., Hubbard, S. R. & Mohammadi, M. Structural basis for FGF receptor dimerization and activation. *Cell* **98**, 641-650 (1999). https://doi.org/10.1016/s0092-8674(00)80051-3
4 Schlessinger, J. *et al.* Crystal structure of a ternary FGF-FGFR-heparin complex reveals a dual role for heparin in FGFR binding and dimerization. *Mol Cell* **6**, 743-750 (2000). https://doi.org/10.1016/s1097-2765(00)00073-3
5 Wang, Y. *et al.* SynQuant: an automatic tool to quantify synapses from microscopy images. *Bioinformatics* **36**, 1599-1606 (2020). https://doi.org/10.1093/bioinformatics/btz760
6 Palmieri, D. *et al.* Her-2 overexpression increases the metastatic outgrowth of breast cancer cells in the brain. *Cancer Res* **67**, 4190-4198 (2007). https://doi.org/10.1158/0008-5472.CAN-06-3316
7 Marquez-Ortiz, R. A. *et al.* IL13Ralpha2 Promotes Proliferation and Outgrowth of Breast Cancer Brain Metastases. *Clin Cancer Res* **27**, 6209-6221 (2021). https://doi.org/10.1158/1078-0432.CCR-21-0361
8 Contreras-Zarate, M. J. *et al.* Estradiol induces BDNF/TrkB signaling in triple-negative breast cancer to promote brain metastases. *Oncogene* **38**, 4685-4699 (2019). https://doi.org/10.1038/s41388-019-0756-z
9 Huang, C., Yuan, P., Wu, J. & Huang, J. Estrogen regulates excitatory amino acid carrier 1 (EAAC1) expression through sphingosine kinase 1 (SphK1) transacting FGFR-mediated ERK signaling in rat C6 astroglial cells. *Neuroscience* **319**, 9-22 (2016). https://doi.org/10.1016/j.neuroscience.2016.01.027
10 Finlay-Schultz, J. *et al.* Breast Cancer Suppression by Progesterone Receptors Is Mediated by Their Modulation of Estrogen Receptors and RNA Polymerase III. *Cancer Res* **77**, 4934-4946 (2017). https://doi.org/10.1158/0008-5472.CAN-16-3541
11 Kabos, P. *et al.* Patient-derived luminal breast cancer xenografts retain hormone receptor heterogeneity and help define unique estrogen-dependent gene signatures. *Breast Cancer Res Treat* **135**, 415-432 (2012). https://doi.org/10.1007/s10549-012-2164-8
12 Wellberg, E. A. *et al.* FGFR1 underlies obesity-associated progression of estrogen receptor-positive breast cancer after estrogen deprivation. *JCI Insight* **3** (2018). https://doi.org/10.1172/jci.insight.120594
13 Bado, I. L. *et al.* The bone microenvironment increases phenotypic plasticity of ER(+) breast cancer cells. *Dev Cell* **56**, 1100-1117 e1109 (2021). https://doi.org/10.1016/j.devcel.2021.03.008
